# Factors Associated with the Prevalence of Thyroid Nodules and Goiter in Middle-Aged Euthyroid Subjects

**DOI:** 10.1155/2017/8401518

**Published:** 2017-03-05

**Authors:** Dalia Dauksiene, Janina Petkeviciene, Jurate Klumbiene, Rasa Verkauskiene, Jelena Vainikonyte-Kristapone, Audrone Seibokaite, Jonas Ceponis, Vygantas Sidlauskas, Laura Daugintyte-Petrusiene, Antanas Norkus, Birute Zilaitiene

**Affiliations:** ^1^Institute of Endocrinology, Medical Academy, Lithuanian University of Health Sciences, Kaunas, Lithuania; ^2^Faculty of Public Health, Medical Academy, Lithuanian University of Health Sciences, Kaunas, Lithuania; ^3^Department of Endocrinology, Medical Academy, Lithuanian University of Health Sciences, Kaunas, Lithuania

## Abstract

The aim of the present study was to determine associations of thyroid hormone levels and different metabolic parameters and anthropometric measurements with volume of nodular and nonnodular thyroid as well as with prevalence of goiter and thyroid nodules in middle-aged euthyroid subjects. *Methods*. The study consisted of 317 euthyroid subjects aged 48-49 from the Kaunas Cardiovascular Risk Cohort study. Thyroid-stimulating hormone (TSH), free thyroxine (FT4), and antithyroid peroxidase antibody (ATPO) levels, as well as anthropometric and metabolic parameters and smoking information, were evaluated. *Results*. In subjects with and without thyroid nodules, thyroid volume correlated with components of metabolic syndrome, body mass index (BMI), smoking, and TSH levels. In the nonnodular thyroid group, thyroid volume was also positively related to serum insulin and HOMA-IR, whereas a negative correlation between thyroid volume and leptin was identified in the nodular thyroid group. The goiter was identified in 12.3% of subjects. Female gender, thyroid nodules, smoking, BMI, and levels of TSH were independent predictors for goiter. Thyroid nodules were found in 31.2% of participants. Female gender, higher TSH levels, and thyroid volume were independent risk factors for thyroid nodules. *Conclusions*. Female gender, thyroid nodules, smoking, BMI, and TSH levels were identified as potential predictors of goiter. Female gender, TSH levels, and thyroid volume predicted the presence of thyroid nodules.

## 1. Introduction

The most common thyroid disorders are thyroid nodules and goiter that develop as a result of the interplay between genetic, environmental, or endogenous factors. Goiter affects approximately 15.8% of the general population and iodine is the main environmental factor that determines goiter prevalence [1]. The prevalence of ultrasound-detectable nodules ranges from 19% to 67% and only 4–7% of thyroid nodules detected by ultrasound are palpable [2]. Although thyroid nodules and goiter have no clinical symptoms in majority of patients, both conditions can be associated with disorders such as endocrine dysregulation, autoimmune thyroid disease, impaired body composition, or various metabolic abnormalities [3]. Thyroid hormones play an important role in coordinating basal metabolic rate and thermogenesis [4]. As shown in previous studies, thyroid hormones, even within normal ranges, may be associated with various markers of unfavorable metabolic profile [5]. Some authors reported that metabolic syndrome (MetS) and its related components, including obesity, insulin resistance (IR), hypertension, dyslipidemia, and impaired glucose metabolism, are associated with morphological abnormalities of the thyroid and may contribute to increased thyroid volume as well as nodule prevalence [6]. Recent studies highlighted the importance of hyperinsulinemia/insulin resistance, the central features of MetS, in thyroid cell proliferation, which manifested as increased thyroid volume and nodule [7, 8].

Women are more likely to have thyroid disorders, including goiter and thyroid nodules, although some authors could not confirm any gender-related associations [9, 10]. Association of thyroid functional and morphological changes with individual risk factors, especially with smoking habits, has been investigated previously. Some authors have found a significant relationship between smoking and increased thyroid volume, a prevalence of goiter, or thyroid multinodularity in areas with iodine deficiency [11].

The aim of the present study was to determine associations of thyroid hormone levels and different metabolic parameters and anthropometric measurements with volume of nodular and nonnodular thyroid as well as with prevalence of goiter and thyroid nodules in middle-aged euthyroid subjects.

## 2. Subjects and Methods

### 2.1. Study Subjects

The study subjects were participants of the Kaunas Cardiovascular Risk Cohort study started in 1977, when a random sample of 1082 Kaunas schoolchildren aged 12-13 years was screened [12, 13]. In 2012, 507 subjects (63.9% of eligible sample) aged 48-49 participated in the survey. Participants using levothyroxine or antithyroid agents (*n* = 19; 3.7%), those having elevated levels of ATPO (presence of ATPO is an early sign of autoimmune thyroid diseases) (*n* = 53; 10.5%), TSH or FT4 levels outside the reference range (*n* = 34; 6.7%), or who did not undergo thyroid ultrasound examination (*n* = 116; 22.7%) were excluded from the analysis. Finally, data of 317 subjects (142 men and 175 women) were analyzed in this study.

The study protocol was approved by the Lithuanian Bioethics Committee (permission number BE-2-30) and written informed consent was obtained from all participants.

### 2.2. Thyroid Morphology

Thyroid ultrasonography was performed by a single physician who was unaware of the patient clinical conditions. The real-time 3D/4D imaging diagnostic ultrasound system Mindray CD-7 with a multifrequency transducer (L12-4 MHz HiRez Linear) was used. Subjects were examined in the supine position with the neck hyperextended. Thyroid volume was calculated according to the formula: width × length × thickness × 0.479 for each lobe. Goiter was determined as a thyroid volume exceeding 18 mL for women and 25 mL for men. Thyroid nodules greater than 3 mm in diameter were registered. In patients with multiple thyroid nodules, the largest diameter of the largest nodule was considered. Thyroid nodules 10 mm or larger in diameter and smaller nodules (6 mm or greater) with suspicious features for malignancy underwent a fine-needle aspiration biopsy. Based on the thyroid nodularity, participants were split into two groups: nodule-free or those with at least one nodule.

### 2.3. Other Measurements

The height of participants, without shoes, was measured to the nearest centimeter with a stadiometer. The body weight of participants was measured to the nearest 0.1 kg with standardized medical scales. Body mass index (BMI) was calculated as weight (kg) divided by squared height (m^2^). Obesity was defined as BMI of 30 kg/m^2^ or higher. Waist circumference (WC) was measured at the midpoint between the lower margin of the last palpable rib and the top of the iliac crest using a stretch-resistant tape. Blood pressure (BP) was measured from the right brachial artery with a standard mercury sphygmomanometer in the sitting position after 5 minutes of rest. BP measurements were performed to the nearest 2 mmHg. Three consecutive BP measurements were taken. The average of these three measurements was used in the analysis. Information on cigarette smoking was collected using a standard questionnaire. Participants were grouped in daily smokers and others.

### 2.4. Laboratory Analysis

Venous blood samples for laboratory analysis were obtained by venipuncture from all patients between 08.00 and 10.00 h after a minimum of 12 h overnight fast. Hormone levels were analyzed by immunoradiometric assay for TSH and radioimmunoassay for FT4 and ATPO using commercial reagent kits of the IZOTOP (Hungary). Insulin and leptin were investigated by immunoradiometric assay using commercial reagent kits of the DiaSource (Belgium) and evaluated using means of *γ*-ray counter Berthold (Germany). Analytical sensitivity of reagents (lowest detection limit) was 0.11 mU/L for TSH, 0.7 pmol/L for FT4, 2 kU/L for ATPO, 0.1 pg/L for leptin, and 1 mU/L for insulin. The coefficients of variation within a set of reagents/between kits (intra/interassays CV) were 4.7/8.6% for TSH, 2.6/5.7% for FT4, 6.7/7.5% for ATPO, 1.8/2.5% for leptin, and 2.1/6.5% for insulin. The laboratory reference values were 10.1–22.0 pmol/L for FT4, 0.27–3.75 mIU/L for TSH, and 4–16 mU/L for insulin. The reference value for ATPO was <78 kU/L. All measurements for lipids and glucose were performed on an automatic analyzer Cobas Integra 400 plus. Serum lipid levels were determined using conventional enzymatic methods. Hexokinase was used for measurement of glucose level.

IR was determined according to HOMA-IR (homeostasis model assessment of IR). HOMA-IR was calculated using the following formula: fasting insulin (mU/L) × fasting glucose (mmol/L)/22.5 [14]. HOMA-IR >2.5 was used for diagnosis of IR.

MetS was determined according to the criteria of the International Diabetes Federation: central obesity defined as waist circumference ≥94 cm for men and ≥80 cm for women plus any two of the following four factors: raised triglycerides level (≥1.7 mmol/L), reduced high-density lipoprotein (HDL) cholesterol level (for men <1.03 mmol/L and for women <1.29 mmol/L), raised blood pressure (systolic BP ≥130 mmHg or diastolic BP ≥85 mmHg, or treatment of previously diagnosed hypertension), and raised fasting plasma glucose (≥5.6 mmol/L or previously diagnosed type 2 diabetes) [6].

### 2.5. Statistical Analysis

Chi-square (*χ*^2^) test was used for analysis of data when variables were categorical. Continuous variables were tested for normality according to the Kolmogorov-Smirnov test. The results were presented as means ± standard deviation (SD) if the distributions were normal or median and interquartile range (IQR) if the distributions did not meet the criteria of normality. Student's *t*-tests (for normally distributed variables) or the Mann-Whitney tests (for nonnormal distributions) were used to compare two continuous variables. For bivariate correlations, Pearson correlation tests (for parametric data) or Spearman correlation tests (for nonparametric data) (*ρ*) were used. Odds ratios (OR) with 95% confidence interval (CI) were calculated using univariate and multivariate binary logistic regression analysis to estimate associations between different variables and goiter or thyroid nodules. Data were analyzed using IBM SPSS Statistics for Mac (V20.0). *p* value less than 0.05 was considered as statistically significant.

## 3. Results

Characteristics of the study population are presented in Table 1. The mean total thyroid volume of participants was 14.2 ± 5.8 mL; it was significantly higher in men than in women (*p* < 0.001). Women had a higher prevalence of thyroid nodules than men (*p* < 0.001). Serum concentrations of TSH and ATPO were similar in both genders, whereas mean levels of FT4 were higher in men than in women. BMI; waist circumference; blood pressure (both systolic and diastolic); and levels of fasting plasma glucose, fasting insulin, and HOMA-IR values were significantly higher in men than in women. Although levels of total cholesterol were similar in both genders, men had significantly higher levels of LDL cholesterol and triglycerides, whereas HDL cholesterol levels were lower. Proportion of current smokers and prevalence of metabolic syndrome were higher in men than in women.

Participants were split into two groups according to the presence or absence of thyroid nodules. In both groups, thyroid volume significantly correlated with components of metabolic syndrome (waist circumference, blood pressure, fasting plasma glucose, and serum lipid profiles), BMI, smoking status, and levels of TSH (Table 2). In the nonnodular thyroid group, thyroid volume was also positively related to serum insulin levels and HOMA-IR, whereas a negative correlation between thyroid volume and levels of leptin was identified in the nodular thyroid group.

We also analyzed the relationship between thyroid size, prevalence of thyroid nodules, and a number of previous pregnancies; however, no significant associations among these factors were determined (data not shown).

The goiter was identified in 12.3% of subjects with similar proportions in men and women (Table 1). Subjects with goiter had a higher prevalence of thyroid nodules (59% versus 27%, *p* < 0.001), lower levels of TSH (median (IQR): 0.9 (0.6) versus 1.3 (0.8) mU/L, *p* < 0.001), higher HOMA-IR values (median (IQR): 2.3 (1.2) versus 2.0 (1.3), *p* = 0.046), and higher BMI (median (IQR): 28.5 (7.8) versus 25.6 (5.9) kg/m^2^, *p* = 0.018) compared with goiter-free subjects. Also the percentage of daily smokers was higher among subjects with goiter than goiter-free subjects (51.3% versus 27.3%, *p* = 0.002). The multivariate logistic regression analysis showed that female gender, thyroid nodules, smoking, BMI, and levels of TSH were independent predictors for goiter. There was a negative association between the presence of goiter and the levels of TSH (Table 3).

Thyroid nodules were found in 31.2% of the study population with the higher frequency in women than in men (Table 1). Of all subjects, 55 (17.4%) had solitary nodules and 44 (13.9%) had multiple nodules. The mean nodule size was 11.2 ± 6.6 mm (range, 3 to 36 mm). The thyroid nodules were divided into two groups by size: smaller than 10 mm and 10 mm or larger. Of all subjects with thyroid nodules, larger nodules (≥10 mm in size) were found in 6.7% of men and 45.3% of women. There was no difference in frequency of thyroid nodules in daily smokers compared with others (*p* = 0.432). Thyroid nodule size was positively correlated with thyroid volume (*ρ* = 0.404, *p* < 0.001). No significant differences were observed in thyroid hormone levels, blood pressure, different metabolic parameters, and BMI between the subjects with larger thyroid nodules (≥10 mm in size) and the subjects with smaller nodules. In the multivariate analysis, female gender, TSH levels, and thyroid volume were independent risk factors for thyroid nodules (Table 3).

In an analysis limited only to a subgroup of subjects with goiter and/or larger thyroid nodules (≥10 mm in size), a negative correlation between thyroid volume and levels of TSH remained significant for both genders (*ρ* = −0.65, *p* = 0.006 in men and *ρ* = −0.27, *p* = 0.009 in women). In men, thyroid volume was also positively correlated with levels of ATPO (*ρ* = 0.66, *p* = 0.006).

Fine-needle aspiration biopsy was performed in 35 patients with thyroid nodules 10 mm or larger in diameter and those with smaller nodules (6 mm or greater) but suspicious ultrasound features of malignancy (microcalcification in 3 subjects). None of biopsied subjects with thyroid nodules were found to have malignant lesions.

## 4. Discussion

Although nontoxic goiter and thyroid nodules are the most common thyroid disorders in adult population, the etiology of both conditions is not completely understood. In this study, we identified some risk factors for goiter and thyroid nodules in middle-aged euthyroid subjects. Female gender, thyroid nodules, smoking, BMI, and lower TSH levels were found to be independently associated with higher risk of goiter. The risk of increased prevalence for thyroid nodules was significantly associated with thyroid volume, female gender, and TSH levels.

A number of earlier studies have analyzed factors that can affect thyroid morphological abnormalities. Iodine undoubtedly plays the most important role in function and structure of the thyroid gland. Epidemiological studies have shown that the abnormality of iodine intake in population may result in thyroid dysfunction and/or abnormal thyroid structure [15, 16]. The best surrogate marker of iodine deficiency in populations is the prevalence of goiter [17]. Studies performed in iodine-deficient areas have demonstrated a negative correlation between iodine intake and thyroid volume, as well as a lower frequency of thyroid enlargement after national iodine intake programs were implemented [18, 19]. Lithuania was considered a low-iodine deficiency area before introduction of iodine fortification [20]. The effectiveness of an iodine prophylaxis program that was started in 2005 is proven by changes in thyroid volume and goiter prevalence [21]. In our study, mean thyroid volumes were lower than those reported in a previous Lithuanian study before iodine supplementation (16.5 mL versus 18.3 mL in men and 12.3 mL versus 16.1 mL in women, resp.) [22].

An association between gender and thyroid volume or goiter prevalence has been reported. Knudsen et al. noted that thyroid volume was larger in men than in women, yet women were 2- to 10-fold more likely to have goiter than men [10]. In our study, men had higher mean thyroid volume compared to women, but the overall prevalence of goiter did not differ between genders. Some authors showed that gender-related differences became evident only after puberty, suggesting that sex hormones may play a role in thyroid volume [23]. Association between thyroid volume and parity status in healthy women has been studied previously [24]. It has been suggested that pregnancy increases thyroid volume, particularly when combined with tobacco smoking and iodine deficiency [24, 25]. We found no significant associations between parity and thyroid volume or prevalence of goiter/thyroid nodules in our study.

Some researchers reported that men had a larger thyroid gland than women due to the difference in body mass [26]. The relationship between thyroid volume and anthropometric measurements (height, weight, body mass index, and body surface area) has been studied extensively. Previous studies have shown that thyroid volume increases with increasing body mass index or body surface area [27, 28]. Our results of significant associations between thyroid volume or goiter and higher BMI are in line with the observations from many other studies [28, 29]. Although BMI does not measure body fat directly, it can be an acceptable indicator of body adiposity [30]. We hypothesize that subjects with higher body weight may have the tendency to accumulate fat in the thyroid tissue resulting in larger thyroid volume. A recent study revealed the link between obesity and morphological changes in the thyroid gland: it was suggested that excessive adiposity lead to expansion of the interfollicular adipose depot or steatosis in thyroid follicular cells (thyroid steatosis) [31]. Furthermore, high BMI leads to hyperinsulinemia and IR that also contribute to increased thyroid cell proliferation and goiter formation. On the other hand, in contrast with reports of other authors [32], we failed to demonstrate an independent association between BMI and thyroid nodules. It is possible that other important growth factors stimulate thyroid cell structural transformation and proliferation independently of BMI. Moreover, complex interactions between environmental and genetic factors are also involved in the etiology of thyroid nodules [33].

Thyroid nodules are 4 times more common in women than men and their frequency increases with advancing age and iodine deficiency [34]. Causes for the higher incidence of thyroid nodules in women are unclear. Both estrogen and progesterone may contribute to the strong female preponderance in thyroid nodular disease. Kung et al. reported that pregnancy was associated with new thyroid nodule formation and an increase in size of preexisting thyroid nodules [35]. Results of the present study showed that female gender is an independent risk factor not only for goiter but also for thyroid nodules, although some authors could not confirm any gender-related associations [9].

TSH plays a major role in regulation of thyroid cell growth and differentiation and may play a direct role in nodule formation [36]. Conflicting data exists concerning the effects of TSH on thyroid growth. Some studies observed significant association between serum TSH and thyroid volume [37, 38], although others did not confirm any TSH-related associations [39], suggesting that TSH alone is not a mitogenic factor [40]. Some authors reported that iodine may modulate the thyroid cell response to TSH [41]. Others suggested that suppressive effect of iodine may enhance the influence of other goitrogenic factors and increase the sensitivity of thyrocytes to TSH in iodine-deficient areas; therefore, normal TSH levels become a goitrogen [23]. The findings of our study revealed an inverse relation of TSH with thyroid volume and goiter. This relation does not seem to be related to the direct effect of TSH on the thyroid gland but may be related to the gradual increase in the production of thyroid hormones in goiter (autonomous thyroid function) or with an increased thyroid mass itself. Another explanation for lower levels of TSH in larger thyroids could be the presence of thyroid-stimulating antibodies. Unfortunately, our patients were not tested for these antibodies. Present study also showed that increased serum TSH is an independent risk factor for thyroid nodules, suggesting that TSH is an important factor in thyroid nodule formation. In the last few years, several reports showed that increased concentrations of serum TSH, even within normal ranges, were associated with a higher frequency of thyroid malignancy and more advanced stage of thyroid cancer [42, 43]. In our study, none of nodules had malignant lesions.

Some authors highlighted importance of MetS and IR in thyroid volume increase and higher risk for formation of thyroid nodules [6, 44]. Rezzonico et al. reported that higher levels of insulin as a growth factor stimulated thyroid cell proliferation [44]. Results of our study also suggested that factors related to MetS may contribute to increased thyroid volume; however, insulin level and IR were associated only with nonnodular thyroid volume. Explanation for this observation could be that hyperinsulinemia and IR have a greater effect on thyroid tissue growth than on thyroid cell structural transformation [44]. However, neither IR nor MetS showed an independent effect in predicting goiter or thyroid nodules. There is no doubt that a variety of other growth factors play an important role in thyroid growth and/or proliferation. It has been previously shown that insulin-like growth factor-1 in cooperation with insulin and TSH stimulates thyroid cell cycle progression and proliferation [45].

Conflicting data exist regarding the relation between tobacco smoking and goiter. Some researchers have found a positive association between smoking and higher prevalence of goiter [46, 47] and/or nodules [47, 48], although others could not confirm any significant smoking-related associations [49]. Differences in iodine intake of the populations may account for such discrepancies [11]. In our study, smoking was associated with higher thyroid volume and was an independent risk factor for goiter. However, we failed to demonstrate an association between smoking habits and thyroid nodules. Fukayama et al. reported that the goitrogenic effect of smoking on thyroid could be caused by thiocyanate, a degradation product of cyanide in tobacco smoke, which competitively inhibits iodine uptake and organification [50].

Our study has some limitations that offer opportunities for future investigations. We acknowledge that a major limitation of our study is a relatively small number of subjects. Moreover, we cannot currently provide data on iodine intake. Studies directly assessing iodine intake in Lithuanian population after introduction of iodized salt are lacking. Therefore, we can only speculate that our subjects are not currently iodine deficient, although they had been exposed to an iodine-deficient environment previously. We also did not analyze effects of selenium, zinc, or other factors that are also important in thyroid metabolism and may affect thyroid function, size, and nodules. Lack of repetitive TSH testing in subjects should be also mentioned as a possible limitation of our study. Repetitive TSH testing may help to obtain a more accurate evaluation of thyroid status and to avoid discrepancies between the results. Another limitation of our study is that we used HOMA-IR for assessment of IR, which is a surrogate marker of IR. The “gold standard” for IR assessment today is the hyperinsulinemic-euglycemic clamp, which is more accurate in evaluating insulin resistance [51].

The strength of the present study includes the use of the data from a randomly selected cohort that has been prospectively followed up for 35 years. A further strength is extensive phenotypic characterization of participants, which includes an elaborate set of anthropometric measurements and metabolic factors, as well as a complete thyroid status assessment with measurements of TSH, FT4, and antithyroid peroxidase antibodies. The narrow age range of subjects eliminates the potential effect of age that may influence the observed associations among variables.

## 5. Conclusions

This study showed that female gender, thyroid nodules, smoking, BMI, and lower levels of TSH are independently associated with an increased risk of goiter. Prevalence of thyroid nodules was found to be significantly associated with female gender, higher levels of TSH, and increased thyroid volume.

## Figures and Tables

**Table 1:** Characteristics of the study population.

Characteristic	Men (*n* = 142)	Women (*n* = 175)	*p* value
Thyroid volume (mL)^∗^	16.5 ± 6.0	12.3 ± 5.0	<0.001
Prevalence of goiter, *n* (%)	12 (8.5)	27 (15.4)	0.06
Prevalence of thyroid nodule, *n* (%)	28 (19.7)	71 (40.6)	<0.001
Thyroid nodule size (mm)^∗^	12.0 ± 3.3	11.2 ± 6.8	0.748
TSH (mU/L)^∗∗^	1.2 (0.7)	1.2 (0.9)	0.436
FT4 (pmol/L)^∗^	15.1 ± 2.1	14.5 ± 2.2	0.005
ATPO (kU/L)^∗∗^	0.05 (1.1)	0.3 (1.1)	0.332
Insulin (mU/L)^∗∗^	9.6 (5.5)	8.9 (3.5)	0.042
FPG (mmol/L)^∗∗^	5.2 (0.8)	4.9 (0.6)	<0.001
HOMA-IR^∗∗^	2.3 (1.4)	1.9 (1.0)	0.004
Leptin (pg/L)^∗∗^	4.1 (4.5)	11.2 (10.7)	<0.001
TC (mmol/L)^∗^	6.0 ± 1.1	6.0 ± 1.1	0.995
HDL (mmol/L)^∗∗^	1.5 (0.5)	1.8 (0.6)	<0.001
LDL (mmol/L)^∗^	3.9 ± 1.0	3.6 ± 1.2	0.036
TG (mmol/L)^∗∗^	1.3 (0.8)	1.0 (0.5)	<0.001
SBP (mmHg)^∗∗^	132.7 (23.3)	126.0 (21.3)	<0.001
DBP (mmHg)^∗∗^	88.7 (13.3)	80.7 (13.3)	<0.001
WC (cm)^∗^	95.8 ± 11.9	85.2 ± 13.8	<0.001
BMI (kg/m^2^)^∗∗^	26.9 (5.4)	25.4 (6.7)	0.076
Obesity, *n* (%)	34 (23.9)	36 (20.6)	0.472
Metabolic syndrome, *n* (%)	44 (31)	32 (18.3)	0.009
Smoking, *n* (%)	59 (41.5)	37 (21.1)	<0.001

^∗^Mean ± standard deviation; ^∗∗^median (interquartile range).

ATPO: antithyroid peroxidase antibodies; BMI: body mass index; DBP: diastolic blood pressure; FPG: fasting plasma glucose; FT4: free thyroxine; HDL: high-density lipoprotein; HOMA-IR: insulin resistance index; LDL: low-density lipoprotein; SBP: systolic blood pressure; TC: total cholesterol; TG: triglycerides; TSH: thyroid stimulating hormone; WC: waist circumference.

**Table 2:** Relationship between thyroid volume and different variables in subjects with and without thyroid nodules.

Characteristic	Thyroid volume			
	Nodular thyroid		Nonnodular thyroid	
	*ρ*	*p* value	*ρ*	*p* value
TSH (mU/L)	−0.43	<0.001	−0.17	0.012
FT4 (pmol/L)	0.12	0.252	0.11	0.099
ATPO (kU/L)	−0.05	0.613	0.02	0.75
Insulin (mU/L)	0.14	0.171	0.21	0.002
FPG (mmol/L)	0.22	0.03	0.27	<0.001
HOMA-IR	0.17	0.086	0.26	<0.001
Leptin (pg/L)	−0.29	0.004	−0.12	0.083
TC (mmol/L)	0.02	0.83	−0.02	0.742
HDL (mmol/L)	−0.3	0.003	−0.37	<0.001
LDL (mmol/L)	0.11	0.275	0.13	0.062
TG (mmol/L)	0.12	0.238	0.24	<0.001
SBP (mmHg)	0.19	0.066	0.14	0.037
DBP (mmHg)	0.29	0.004	0.14	0.036
WC (cm)	0.33	0.001	0.41	<0.001
BMI (kg/m^2^)	0.22	0.026	0.3	<0.001
Smoking	0.3	0.003	0.21	0.001

ATPO: antithyroid peroxidase antibodies; BMI: body mass index; DBP: diastolic blood pressure; FPG: fasting plasma glucose; FT4: free thyroxine; HDL: high-density lipoprotein; HOMA-IR: insulin resistance index; LDL: low-density lipoprotein; SBP: systolic blood pressure; TC: total cholesterol; TG: triglycerides; TSH: thyroid-stimulating hormone; WC: waist circumference; *ρ*: correlation coefficient.

**Table 3:** Multivariate analysis of associations of goiter and thyroid nodules with analyzed factors.

Variable	Goiter		Thyroid nodule	
	OR (95% CI)	*p* value	OR (95% CI)	*p* value
TSH (mU/L)	0.09 (0.03–0.26)	<0.001	1.61 (1.05–2.45)	0.028
FT4 (pmol/L)	1.12 (0.92–1.38)	0.271	0.95 (0.83–1.07)	0.388
ATPO (kU/L)	1.03 (0.99–1.06)	0.166	0.99 (0.97–1.01)	0.355
BMI (kg/m^2^)	1.26 (1.1–1.44)	0.001	0.94 (0.86–1.02)	0.151
Metabolic syndrome	0.72 (0.23–2.22)	0.568	1.02 (0.45–2.28)	0.968
Leptin (pg/L)	0.93 (0.86–1.003)	0.06	1.02 (0.97–1.08)	0.378
HOMA-IR	0.91 (0.64–1.3)	0.618	0.91 (0.7–1.19)	0.478
Smoking	3.91 (1.68–9.06)	0.002	0.83 (0.45–1.54)	0.557
Women versus men	4.46 (1.46–13.61)	0.009	4.29 (1.98–9.31)	<0.001
Thyroid nodule	4.13 (1.82–9.38)	0.001		
Thyroid volume (mL)			1.16 (1.09–1.23)	<0.001

ATPO: antithyroid peroxidase antibodies; BMI: body mass index; FT4: free thyroxine; HOMA-IR: insulin resistance index; TSH: thyroid-stimulating hormone.
