# Corrigendum to “Factors Associated with the Prevalence of Thyroid Nodules and Goiter in Middle-Aged Euthyroid Subjects”

**DOI:** 10.1155/2019/9131406

**Published:** 2019-01-29

**Authors:** Dalia Dauksiene, Janina Petkeviciene, Jurate Klumbiene, Rasa Verkauskiene, Jelena Vainikonyte-Kristapone, Audrone Seibokaite, Jonas Ceponis, Vygantas Sidlauskas, Laura Daugintyte-Petrusiene, Antanas Norkus, Birute Zilaitiene

**Affiliations:** ^1^Institute of Endocrinology, Medical Academy, Lithuanian University of Health Sciences, Kaunas, Lithuania; ^2^Faculty of Public Health, Medical Academy, Lithuanian University of Health Sciences, Kaunas, Lithuania; ^3^Department of Endocrinology, Medical Academy, Lithuanian University of Health Sciences, Kaunas, Lithuania

In the article titled “Factors Associated with the Prevalence of Thyroid Nodules and Goiter in Middle-Aged Euthyroid Subjects” [1], an acknowledgment should be added as follows: “This research was funded by a grant no. SEN-14/2015 from the Research Council of Lithuania.”
